# Acceptability of participatory social network analysis for problem-solving in Australian Aboriginal health service partnerships

**DOI:** 10.1186/1472-6963-12-152

**Published:** 2012-06-10

**Authors:** Jeffrey Fuller, Wendy Hermeston, Megan Passey, Tony Fallon, Kuda Muyambi

**Affiliations:** 1 School of Nursing & Midwifery, Flinders University, GPO Box 2100, Adelaide, SA 5001, Australia; 2 Social Policy Research Centre, University of New South Wales, Sydney, Australia; 3 University Centre for Rural Health – North Coast, University of Sydney, Lismore, Australia; 4 Centre for Rural Health and Community Development, University of South Australia, Adelaide, Australia

## Abstract

**Background:**

While participatory social network analysis can help health service partnerships to solve problems, little is known about its acceptability in cross-cultural settings. We conducted two case studies of chronic illness service partnerships in 2007 and 2008 to determine whether participatory research incorporating social network analysis is acceptable for problem-solving in Australian Aboriginal health service delivery.

**Methods:**

Local research groups comprising 13–19 partnership staff, policy officers and community members were established at each of two sites to guide the research and to reflect and act on the findings. Network and work practice surveys were conducted with 42 staff, and the results were fed back to the research groups. At the end of the project, 19 informants at the two sites were interviewed, and the researchers conducted critical reflection. The effectiveness and acceptability of the participatory social network method were determined quantitatively and qualitatively.

**Results:**

Participants in both local research groups considered that the network survey had accurately described the links between workers related to the exchange of clinical and cultural information, team care relationships, involvement in service management and planning and involvement in policy development. This revealed the function of the teams and the roles of workers in each partnership. Aboriginal workers had a high number of direct links in the exchange of cultural information, illustrating their role as the cultural resource, whereas they had fewer direct links with other network members on clinical information exchange and team care. The problem of their current and future roles was discussed inside and outside the local research groups. According to the interview informants the participatory network analysis had opened the way for problem-solving by “putting issues on the table”. While there were confronting and ethically challenging aspects, these informants considered that with flexibility of data collection to account for the preferences of Aboriginal members, then the method was appropriate in cross-cultural contexts for the difficult discussions that are needed to improve partnerships.

**Conclusion:**

Critical reflection showed that the preconditions for difficult discussions are, first, that partners have the capacity to engage in such discussions, second, that partners assess whether the effort required for these discussions is balanced by the benefits they gain from the partnership, and, third, that “boundary spanning” staff can facilitate commitment to partnership goals.

## Background

Health care for chronic conditions in cross cultural settings is often complex. One response of health services has been to form cross cultural health and community service partnerships [1-5]. In Australia the most pressing issues in cross cultural health care pertain to Aboriginal and Torres Strait Islander communities [6,7] and so partnerships have been formed between Aboriginal community-controlled health services and health services that are provided for the whole community (mainstream health services) [8-12]. In Aboriginal and Torres Strait Islander health, however, little work has been done to critically assess how such partnerships function, particularly at the level of service delivery involving front-line health workers [13-17].

Service partnerships differ according to their location because of the unique and complex interactions between stakeholders and the particularities of local health care contexts. Hence, specific strategies to change service partnerships in one local area and for particular cultural groups may not be applicable elsewhere. The general problem-solving methods used by partnership developers may well be generalisable, however, and so these can be transferred into policy [18]. These methods may offer a means to implement partnership principles, such as those suggested by the Closing Gap Steering Committee for Indigenous Health Equality: that parties should be willing to negotiate; to have a sustained commitment to ongoing review; and be open to new ways of working that might involve compromise and cultural change [17].

There is an extensive literature on the use of participatory problem solving methods in health and human services, with increasing use of a range of different techniques with new computer applications [19,20]. The value of adding social network analysis to participatory processes, as distinct from other participatory modelling techniques, is that network links between members can be shown in explicit detail from empirical data that is generated from all of the members’ individual input. A map of these network links can be displayed visually, enabling ready interpretation of network features by members [21].

Hence, with increasing attention to a network approach to the organisation of health services [22-24] there is value in exploring participatory social network analysis as a process through which network members can solve problems in their partnerships [21,25]. When network data are fed back to members they can see how the partnership functions as a network and with these data they can engage in joint network problem-solving. While there is some research about the use participatory social network analysis in cross-cultural settings [21], this has not addressed acceptability issues per se. Studying the application of participatory social network analysis in Australian Aboriginal health will increase understanding about its application and acceptability in this cross-cultural context.

In Aboriginal health, participatory approaches offer a difference to the researcher-driven descriptive approaches, which have failed to provide evidence about the strategies needed to improve Aboriginal and Torres Strait Islander health [26-28]. Participatory approaches require stakeholder engagement in research and so build research capacity as one product of the research act [29,30], however the nature of participatory network analysis can be confronting. For instance explicit network data that is fed back could be sensitive to some and the identity of individuals or organisations might be inferred. While ethical guidelines have been developed to minimise such risks [31], these sensitivities and risks may be exacerbated in cross cultural contexts where there are differences in styles of communication and in dealing with conflict. For instance cultural groups with a collectivist community orientation have been found to use more integrating and compromising style of conflict communication than groups with a more individualistic orientation [32]. Power imbalances between groups, particularly for colonised people such as Australian Aboriginal and Torres Strait Islanders, have been found to act as a barrier to effective communication and participation in health service activities [33]. In a study of partnership readiness for participatory research, Andrews et al concluded that this required goodness of fit and capacity [34]. They defined goodness of fit as compatibility and mutual interest within a climate that is conducive and based on participants’ history and prior knowledge of each other. Clearly prior knowledge requires time and up to three years has been reported as needed to develop community ownership in partnership projects with university researchers [35]. Hence the use of participatory social network analysis with explicit display of people’s position in a network and subsequent group discussion about this could be too direct and counter to communication norms and history of partnerships for some groups [36]. Bearing these issues in mind, the aim of this study was to determine whether a participatory research process involving social network analysis is acceptable for problem-solving in cross-cultural partnerships in Australian Aboriginal health care.

## Methods

### Setting

Two participatory case studies were conducted over 18 months between 2007 and 2008 in two Australian states. Each case involved an existing partnership between Aboriginal community-controlled and mainstream health care services for improving the local service response to chronic disease. The two sites were chosen because they were known to the researchers and the service managers expressed an interest in the method. For a description of the two sites see Table 1 and for the sequence of case study activities see Table 2.

**Table 1:** Partnership types and participants

	**Site A**		**Site B**	
Network type	Interagency network of one Aboriginal Medical Service and three mainstream health service organisations.		Hub-and-spoke service model with a travelling clinic for four Aboriginal communities; clinics coordinated from the regional hub.	
Partnership origin	Joint development by the Aboriginal Medical Service and mainstream area health service in 2003 in response to State Government funding.		Started in 2004 under a State Primary Health Care Initiatives grant focused on collaboration between agencies that provide services to local Aboriginal communities.	
Governance processes (before the research project)	Partnership formalised through a memorandum of understanding.		Management centred on a clinic coordinator and an earlier clinic management committee that had ceased to meet.	
	Governance structures comprise: (1) a management committee of service managers and a representative from the local Aboriginal Health Advisory Council; and (2) a committee of service providers drawn from the partners who developed the service operations. Both committees have terms of reference.		No written agreement between the partners.	
			No partnership meeting or review processes in place.	
Dedicated partnership staff	Partnership program manager and a part-time project officer employed by the mainstream area health service.		A clinic coordinator employed by the mainstream area health service.	
Staff in the network	Aboriginal health workers, mainstream mental health service providers and service managers; employed in either one of the four organisations.		Aboriginal health workers and health education officers, medical officers, nurses and allied health clinic staff, and service managers; employed by the Aboriginal Medical Service, the mainstream area health service or as private practitioners.	
Survey and focus group participants	Identified (21)	Surveyed (20)	Identified (29)	Surveyed (22)
	· 5 Aboriginal	· 4 Aboriginal	· 10 Aboriginal	· 5 Aboriginal
	· 16 non-Aboriginal	· 16 non-Aboriginal	· 19 non-Aboriginal	· 17 non-Aboriginal
				Focus groups (9)
				· 9 Aboriginal
Key informants	Identified (13)	Interviewed (11)	Identified (10)	Interviewed (8)
	· 6 Aboriginal	· 4 Aboriginal	· 6 Aboriginal	· 5 Aboriginal
	· 7 non-Aboriginal	· 7 non-Aboriginal	· 4 non-Aboriginal	· 3 non-Aboriginal

**Table 2:** Sequence of case study activities

**Site A**	**Site B**
First Local Research Group Meetings: establish the project	
Network and team function survey	
	Focus groups to include Aboriginal members who did not want to be surveyed
Second Local Research Group Meetings: review data and commence problem solving	
Three special problem solving workshops conducted	Management meetings recommenced
	Three meetings held
Third Local Research Group Meetings: review progress and next steps	
Key informant interviews and critical researcher reflections: ascertain the acceptability of the method	

### Local research groups

A local participatory research group was established at each site to guide the research and to consider and act on the findings. The groups comprised Aboriginal and mainstream staff from each service in the partnership, Aboriginal and health system policy officers, Aboriginal community members and the researchers. Three meetings were scheduled at each site, involving 13–19 participants per meeting. Additional meetings were held as required to deal with particular partnership issues that arose at each site. The discussions in the groups were facilitated by at least two of the researchers. The post-survey meetings were facilitated to discuss the following:

•Which workers have the most links with other workers and how could these be built upon?

•Are there opportunities to build more links between some workers?

•What challenges for the partnership are revealed by the survey and what can be done to address them?

The discussions were audio recorded. Facilitating researchers made summary thematic notes describing the issues that were raised and took minutes of the group decisions and the proposed actions.

### Survey

A network and work practice survey was administered by face-to-face interviews at each site with 42 partnership staff to ascertain the network structure of the partnership and staff perspectives about the purpose and roles of the partnership. Interviews were considered by the local research groups to be a more culturally secure approach to data collection than a postal survey.

For the network component of the survey, we used the social network analysis technique described by Fried [37] and by Provan [21]. Four prominent service providers and managers from different sectors at each site were purposively selected for their breadth of knowledge about the local service partnership. They each listed the workers whom they considered made up the local service network and we combined the four lists to create a final composite list for each site. All staff on the composite lists were approached to participate in the survey about their links with all other listed workers in relation to the exchange of clinical information, the exchange of cultural information, team care, management and planning, and policy development. The survey questions and definitions of what constituted a link were provided to participants in order to maximise clarity and consistency about what was being asked (see Table 3).

**Table 3:** Survey questions and link definitions

**Question**	**Link definition**
*1a. Who do you give clinical information to?*	Clinical information is defined as the exchange of information about the client’s condition/illness and the treatment and care of that condition.
*1b Who do you receive clinical information from?*	
*2a Who do you give cultural information to?*	Cultural information is defined as exchange of information about the customs of Aboriginal people (identity, habits, language and communication, laws and morals, connections to land, family and community).
*2b Who do you receive cultural information from?*	
*3. Who do you undertake team care with?*	Team care is defined as joining with other workers to provide health care.
*4. Who do you undertake management and planning of services with?*	Management and planning of services is defined as joining with other workers about the organisation of resources (staff, funding, equipment) and the development of strategies so that services can achieve their goals.
*5. Who do you undertake policy development with?*	Policy development is defined as joining with other workers in the negotiation and preparation of statements at local, regional and statewide level.

We analysed the worker links and overall network density (an indicator of how many workers are linked) to determine the structure of the network linkages between particular workers, as well as to compare the overall level of connectivity among workers for each activity.

For the work practice component of the survey, we used the ‘work practice questionnaire’ and the ‘team climate inventory’ [38,39]. The work practice questionnaire measures individual, team and organisational factors that influence health care work practices; its construct and criterion validity have been established [38]. We adapted eight of the questionnaire scales to the foci at the two sites: mental health and diabetes. The team climate inventory is a well-publicised, psychometrically robust, five-factor instrument applicable for group-level analysis of team vision, participant safety, support for innovation, task orientation and interaction frequency [39]. The Likert-type items on the scales of both the questionnaire and the inventory were described as means and 95% confidence intervals. The results were fed back as a simple display of the items on each scale that scored highest and lowest, which were those items for which the confidence intervals did not cross. Table 4 gives an example of high and low scoring items on four of the scales.

**Table 4:** Examples of items from the work practice survey at site B

**Item**	**High level agreement**	**Mean (max)**	**Lower level agreement**	**Mean (max)**
Team vision (team climate inventory)	The goals of the program are worthwhile to the Aboriginal community.	6.2 (7)	The goals of the program are clearly understood, and these goals can be achieved.	4.9 (7)
Task orientation (team climate inventory)	Program workers are concerned about achieving the highest standards of performance.	5.0 (7)	The program evaluates potential weaknesses to achieve best possible outcomes.	3.8 (7)
Interaction frequency (team climate inventory)	Staff keep in regular contact.	3.3 (5)	Team members have frequent formal meetings.	2.5 (5)
Team work factors (work practice questionnaire)	The skills of the team mean that the team is well equipped to respond.	3.7 (4)	Morale is high among the team.	3.2 (4)
	In general, team members have good relationships with other program staff.	3.7 (4)		

Separation into higher and lower level agreement on items for which the confidence intervals did not cross.

These data were provided to LRG members who were not trained in social network methods. In order to increase understanding we used lay terms where possible rather than technical network terms. An example of this was to use the term “bridging” rather than “betweenness” to describe the intermediary function of a worker who occupied a critical junction position in the network.

### Focus groups

Some Aboriginal health staff at site B were reluctant to take part in the survey because of anonymity concerns and a preference for a more narrative and story-telling approach. Recognising their concerns, two focus groups involving nine Aboriginal staff were conducted by an Aboriginal research team member, thus increasing the participation of Aboriginal partnership members. The focus group discussion centred on the value of the partnership, the participants’ current roles and their hopes for their future role.

### Key informant interviews

Towards the end of the project, we audio recorded semi-structured interviews with 19 key informants at both sites. These included managers, service delivery staff and health policy officers. The interviews sought their views about the value of the participatory process in helping each partnership to solve problems. The prompts included:

The usefulness of the participatory process and the information it generated

Current and anticipated changes to the partnership as a result of the participatory process

The extent to which the process enabled voices to be heard and facilitated input to the partnership

The cultural and methodological safety of the research process

Detailed de-identified notes were returned to each informant for verification and further comment and were then divided between two of the researchers for thematic analysis. Data were first coded under themes reflecting the prompts and then under additional themes derived iteratively during reading of the notes. Coding and thematic analyses were compared regularly between the researchers to optimise consistency and to enrich interpretive insights. A draft report of the thematic analysis was written, incorporating the interviews at both sites. The full research team discussed and added insights to the draft report before it was reported back to the local research group at each site.

### Critical researcher reflections

Detailed minutes of local research group and research team meetings were used to document the research process and to inform the researchers’ critical reflections on the method, together with the literature on participatory research and network analysis.

### Ethical issues

Approval was obtained from ethics committees of the University of Sydney, North Coast Area Health Service, New South Wales Aboriginal Health and Medical Research Council and the Aboriginal Health Council of South Australia. Informed consent was obtained from each local research group, survey and interview participants. A set of local research group norms relating to cultural safety and respect were established to guide the discussion of the findings.

Anonymity was difficult to guarantee in these participatory case studies of relatively small organisational partnerships, even though we used codes and broad worker categories on network maps. Participants were informed about how the data would be displayed and told that sensitive findings would be discussed with them before reporting. Data from Aboriginal participants were collected either by a research officer who was Aboriginal or someone who had worked with that community and with whom the participants felt comfortable.

## Results

The characteristics of each case study site and the numbers of survey participants and key informants are listed in Table 1.

### Survey, focus groups and LRGs

The overall response rates to the survey at the two sites were good (95% and 76%), although only half of the identified Aboriginal workers at site B agreed to participate for the reasons mentioned above.

**Figure 1:**
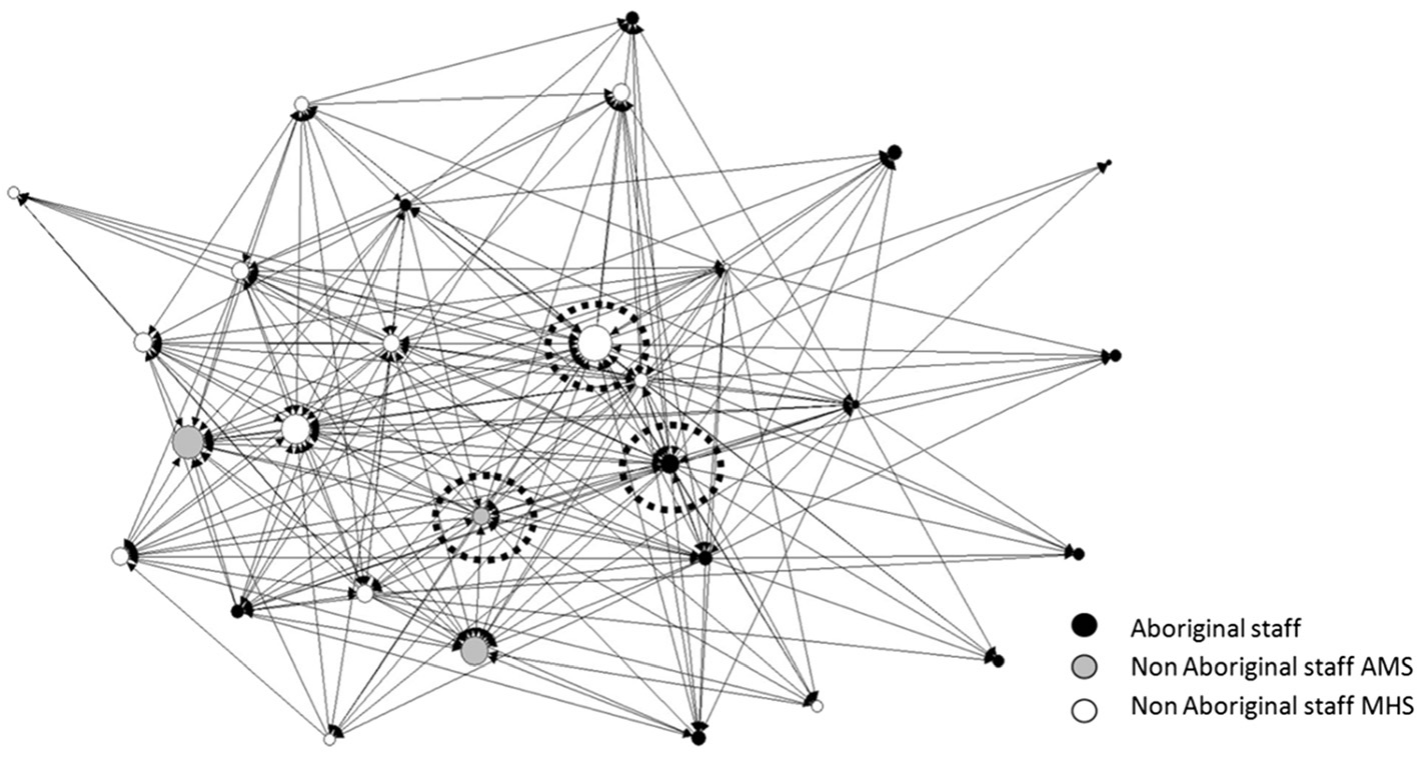
**Team care links at site B.** Directed arrows indicate those who undertake team care with that worker, with node size adjusted to the number of links. Broken line “surround” indicates those workers with the highest betweenness score

Some of the mapped and tabulated data for site B are provided as an illustration of the type of information fed back to the local research groups. The map of site B team care links (Figure 1) shows two aspects of these data. First, the workers with the largest number of direct links (larger degree centrality = larger node size) were the Site B Coordinator, another non-Aboriginal worker in the mainstream health service (MHS) and two non-Aboriginal workers in the Aboriginal Medical Service (AMS). Second, the workers who performed the main bridging functions (large node betweenness centrality shown with dotted surround) were the workers who occupy critical positions that join workers in different parts of the network. These bridging workers would have the greatest impact in breaking up the whole network if they were not present. The workers who occupied the main bridging positions on the team care network at site B were in order, the site B Coordinator, an Aboriginal Health Worker from the AMS and a non-Aboriginal worker from the AMS. Through visualisation of these data on maps we were able to show that the network of workers linked by the exchange of team care was highly centred on the site B Coordinator, both in terms of direct links with others, but also as a bridging person. The network of workers linked by the exchange of management and planning in the partnership was also highly centred both in degree centrality and betweenness centrality on this site B Coordinator (map not shown).

The tables provide summary measures of the different types of links. The main finding from the data in Table 5 was a dense network of workers linked on the exchange of team care but a much less dense network of workers linked on the exchange of management and planning or policy development. Table 6 shows that non-Aboriginal workers had significantly more links than Aboriginal workers in relation to the exchange of clinical information and engagement in team care. Aboriginal workers were significantly more highly linked with other partnership members as providers of cultural information.

**Table 5:** Mean links per activity Site B

**Activity**	**Mean**
Clinical information	
· *Given to*	7.05
· *Received from*	7.18
Cultural information	
· *Given to*	4.18
· *Received from*	5.45
Team care	11.80
Management and planning	2.83
Policy	1.54

**Table 6:** Mean links by worker type at site B

**Activity**	**Aboriginal (n = 11)**	**Non-Aboriginal (n = 18)**	***p***
Clinical information			
· *Given to*	3.27	6.61	0.003*
· *Received from*	3.00	6.94	0.001*
Cultural information			
· *Given to*	2.27	3.72	0.065
· *Received from*	7.45	2.11	< 0.001*
Team care with	6.73	10.39	0.006*
Management and planning with	2.09	3.39	0.07
Policy with	1.09	1.11	0.94

Two-sided *t* test for independent samples (unequal variances).

The work practice survey at site B showed a high level of agreement that the goals of the partnership were worthwhile and that workers sought to achieve the highest standards. Agreement was lower, however, that the goals were clearly understood or that the partnership undertook evaluation or held formal meetings (see Table 4). There was agreement that staff had adequate skills and good relationships but less agreement that team morale was high. These findings add to the network analysis, suggesting that the partnership could ensure a more highly linked service in team-based care by adding formal meetings and evaluations within management and planning, for which there were a lower number of links.

LRG and focus group participants confirmed that the survey identified the network role position of different workers accurately and thereby illustrated the network features of the partnerships. Through discussion at the LRGs, current roles of Aboriginal workers were identified at both sites. At site A, these included demands for crucial cultural information from other team members when Aboriginal clients presented to a service. The consequence of this demand was recorded in field notes by one of our research staff:

"The CEO mentioned that the Aboriginal staff had been under a lot of pressure from the community, and in recent months the team had operated under its normal strength due to staff movements. The Aboriginal staff had presented a letter to the partnership management meeting stating that they were withdrawing support for the partnership because its objectives had been developed from the top down and had not involved them and because they were not resourced as the partnership cultural advisors."

"Site A research officer field notes, November 2007."

The lower number of links of the Aboriginal workers with regard to clinical information exchange and team care prompted discussion about their greater role in clinical care, as evident from the following comment made by an Aboriginal worker during one of the site B focus groups:

"[We] fellas are the best people to talk to our people. They open up to us … I think with one of our people sitting with the [doctor], and if we get a bit more out of them then she can make a good assessment … and we can explain to them after [she leaves] what the doctor was talking about."

Hence, one of the challenges identified in the meetings at site B was how to develop their clinical role:

"The group stated that the information shows the workers are doing what is consistent with their current roles but the challenge now is how to get more Aboriginal people involved in clinical roles in case conferences and to have a greater set of clinical skills."

"Site B 2^nd^ local research group notes"

"The Aboriginal Medical Service CEO stated that he now knew that he had to increase the understanding of Aboriginal staff about the clinic and their involvement in it."

"Site B Project Steering Committee Meeting notes Sept 2007."

Another challenge identified from the survey at site B was to create a shared clinical governance process that increased Aboriginal involvement in coordination. At site A, the challenges identified were to strengthen team-based care, improve worker morale in all the teams, and re-engage all of the teams to the objectives of the partnership. Discussion about engagement at both sites centred on partnership purpose, who drove it and who benefited most from it. A project worker at site A saw a need for ongoing re-engagement:

"The biggest challenge if the partnership is to be a success and sustainable - there needs to be a revisit, a continual review of the ownership to ensure that at all stages all parties buy into the objectives. That all parties are given the opportunity to talk about areas where they feel they are not happy … that factor alone will determine the success or failure of the partnership."

### Key informants

For this paper, we coded the data under the themes: “put the network issues on the table”, “opened up problem-solving communication” and “suitability of the method”.

#### Put the network issues on the table

Informants’ responses suggested that the survey data provided a reasonably accurate description of the linkages among different workers in the service networks at both sites. The structure of the networks was revealed by the collective input of all the members, and this understanding incited network members to change certain aspects. As an Aboriginal manager at site B said:

"There appeared to be considerable impact of the maps in leading [managers] to look at the structure of the network, because this was put on the table … because it set out where we were communicating and where we needed to communicate better … so it has set a platform for where we are going with it now."

Most notably, the place of Aboriginal workers in the network of links on clinical and cultural information exchange was “put on the table”, giving these workers information on which to base their concern in further discussions. Putting issues on the table did not mean that the partnership issues were previously unknown but rather they were now publicly acknowledged. For a mainstream service manager at site B, putting partnership issues “on the table” meant that her private concerns were validated, which gave her strength to act:

"[The findings] said it was not just me thinking this. It validated my feelings, my own observations I had made previously. That gave me some strength, some validity to start to address the issues … It opened up the conversation I think, it gave it a framework."

#### Opened up problem-solving communication

Putting issues on the table through the participatory research approach led to self-examination, which in turn opened up problem-solving communication. Communication at both sites was focused on the purpose of the partnership and appropriate worker roles, as illustrated in the following comment of a non-Aboriginal project worker at site A:

"There is a certain level of openness… a debate … which was not there (before). There's been a voice added for Aboriginal workers to express themselves… Because of the open discussion and because of the enhanced appreciation of the other side's point of view, there's been a review of service delivery processes… where some flexibility has been introduced, especially the area around home visits to clients and flexibility around the appointment system."

In relation to roles, the Aboriginal Medical Service CEO at site B reported that the process had provided evidence that Aboriginal workers wanted to take on a greater clinical role, including acting as a broker between clinicians and community members so that clinical communication was effective. Both Aboriginal and non-Aboriginal service providers asked that Aboriginal workers be trained to take this on, and during the project changes were being made to enable such training.

#### Suitability of the method

While the value of the participatory network research was to “put issues on the table” and so open up problem-solving, we were concerned that any findings perceived as reflecting negatively on the network could exacerbate conflict. While informants acknowledged this, there did seem to be acceptance that revealing problems was necessary, as an Aboriginal policy officer at site B stated:

"Meetings got heated at times but … if [the project] had not come along it would have been a lot worse, because you would have had this division between the two teams, with one doing it the Aboriginal way and the other doing it the non-Aboriginal way. It [the research] didn’t have the answers, it was up to the teams to work out what next."

Some informants indicated that the maps revealed the issues as structural, so that subsequent discussion could be framed in terms of network process, “without this getting personal” or related to an individual. The confronting aspects of some of the findings and subsequent discussion were described at site B as “the difficult discussions that we had to have”. Informants at both sites affirmed that most partners continued to engage in discussions, thereby signifying their commitment to working together and to make improvements.

Another indication of the suitability of the method is the extent to which participants have used the results to improve the services. At both sites, informants reported that the following changes were being made at the time the project closed: formation of decision-making groups to act on the findings, increasing the clinical role of Aboriginal workers, partnership agreements finalised at site A and being drafted at site B, proposed coordination changes at site B, and a commitment to include the service partnership in staff work plans at site A. Lack of support for the most central staff member at site B was identified as a potential threat, as the network data suggested that the partnership would suffer if this person were to leave. Consequently, changes were made to the coordination structure at site B, and strategic meetings between service managers were resumed.

## Discussion

In our critical reflection on our experiences and on the literature in the use of the participatory research, we identified three conditions in a service partnership that are conducive to the use of participatory research incorporating network analysis: the capacity of participants to solve partnership problems, perception of the network as beneficial by the participants and the function of “boundary spanners” (key intermediaries) to facilitate trust and reciprocity.

Capacity in partnerships is influenced by the distribution of responsibilities and resources, and hence power. The social network analysis clearly showed which responsibilities were placed on network members and who held network power. The participatory approach then provided a means for Aboriginal staff to voice their concerns about network responsibilities and their roles. In cross-cultural situations, the difficulty that clinical staff have in sharing health decision-making and differences in western and Indigenous negotiation and policy-making can make it more difficult to sort out the responsibilities of partners and to distribute resources and power [19,40,41]. Additional time and resources will be needed for cross-cultural partnerships to develop and to counter embedded power differences [15,17]. It is not surprising, therefore, in an 18-month project, that not all of the partnership issues that had been “put on the table” were resolved, indicating that the timeframe was too short for participatory cross cultural research [42]. A second survey would have been useful to identify any changes in network and team function.

One of the site A teams withdrew from the partnership, explaining that this was because of too great a workload (inadequate capacity), lack of ownership of the partnership objectives and insufficient perceived benefit to them from the partnership. The importance of assessing capacity and benefit has been identified as one of the principles of Indigenous–mainstream partnerships and also in community-based participatory research more generally [17,34]. Ford and colleagues stated that one of the first (and often ongoing) assessments that network partners make is whether to join and then remain in the network [43]. This assessment concerns the “payoff” function, which is an estimate of the costs and benefits of participating in a network. This involves ascertaining whether others share the mutual purpose and commitment and will contribute to the network. Ford and colleagues argued that network analysis can help members to determine their payoff function, because they can situate their position relative to others in the network and can estimate their relative network transaction costs and benefits. The assumption that we tested in this participatory research was that partners would use the network information to make improvements. We can now conclude that a member that has some doubt about the costs and benefits of a network might use network information to decide to leave, rather than stay and make improvements.

In network theory, a payoff assessment is positive when a member trusts others to maintain their commitment to agreed partnership goals and to reciprocate by helping each other [44,45]. An important resource for facilitating the development of trust and reciprocity is a “boundary spanner” [46]. Effective boundary spanners are key network members who generally have good links with others in the partnership but who also have power to influence their home organisation’s commitment to partnership goals [47,48]. Our network analysis clearly revealed the boundary spanning roles at both sites. For instance, at site A, the leader of the Aboriginal team was a boundary spanner who communicated lack of payoff for the team. As no decision was taken on how to address this assessment, then trust and reciprocity within the wider network was threatened. Thus, a boundary spanner can significantly influence the partners’ preparedness to solve problems, particularly as compromise and cultural change have been suggested as important for partnership success [17].

In addition to the conditions in a partnership that are conducive to the use of participatory network analysis, we also learnt about the need for flexibility. Our decision to use focus groups in response to the concern of some Aboriginal staff about the potential revelation of identities in the network survey indicated our recognition of the lack of cultural security in our original approach.

One strength of the study was the use of multiple methods for data collection and for validation and enrichment of interpretation by participant feedback, techniques known to be useful for qualitative and participatory research [49,50]. Our ability to draw firm conclusions from these two case studies about the use of cross-cultural participatory network analysis is, however, limited, and the method should be tested in further cross-cultural contexts.

## Conclusion

Participatory network analysis can be used in Aboriginal–mainstream health service partnerships to improve services. In relation to the research question (“Is a participatory research process incorporating network analysis an acceptable problem-solving process in Aboriginal–mainstream primary health care partnerships?”), we found that the power of the process was that it “put the issues on the table”. Borgatti and Molina warned, however, that this feature requires caution [51], as findings can be confronting when interpreted as reflecting negatively on the network or on its members. Hence, researchers using this approach must ensure that adequate problem-solving processes and timeframes are built into the participatory design. We found three conditions that were conducive to the use of participatory network analysis for partnership improvement: capacity to solve problems, perception of network benefit, and the role of boundary spanners. Further research would indicate how to engage health service workers in a partnership in network analysis, so that they are prepared for what it will entail and appreciate the potential benefits of working on issues that “are put on the table”. Although data feedback is important, without other strategies it does not itself lead to change [52].

Our findings show how participatory social network analysis can be used to support partnership problem-solving in a way that addresses the ethical risks [21,25,51]. Our study has identified that participatory social network analysis was acceptable in these two Aboriginal-mainstream cross cultural health service settings. While there were differences in positions of power between the Aboriginal and mainstream workers, their respective capacities and mutuality of interest in being in a partnership, these differences did not preclude its use. In fact, with the adaptation of methods to accommodate differences in preferred communication styles and the need for cultural safety, the participatory approach did enable the Aboriginal health workers to have a voice and in so doing gave them some power. If participatory social network analysis can be conducted ethically, with flexibility and with sufficient rigour that the findings adequately represent the network, then this approach can provide valuable data for reflective partnership practice and serve as a generalisable problem-solving process for primary health care partnerships. This was the case in the particular cross cultural Aboriginal health contexts of this study, however further studies with other groups would validate its broader cross cultural acceptability.

## Competing interests

The authors declare that they have no competing interests.

## Author contributions

JF, WH and MP designed the study. All the authors were involved in the conduct of the study, including data collection and analysis. TF wrote first drafts of sections of the paper and JF, WH and MP wrote the later drafts. All the authors contributed during drafting, and all authors read and approved the final manuscript.

## Pre-publication history

The pre-publication history for this paper can be accessed here:

http://www.biomedcentral.com/1472-6963/12/152/prepub
